# Lipid on stroke in intracranial artery atherosclerotic stenosis: a mediation role of glucose

**DOI:** 10.3389/fendo.2024.1322114

**Published:** 2024-08-20

**Authors:** Sheng Li, Yiqing Wang, Xiangyi Zhu, He Zheng, Jianqiang Ni, Hao Li, Yi Yang

**Affiliations:** ^1^ Departments of Neurology, The First Affiliated Hospital of Soochow University, Suzhou, Jiangsu, China; ^2^ Department of Critical Care Medicine, Suzhou Wuzhong People’s Hospital, Suzhou, Jiangsu, China; ^3^ Department of Neurology, The Fourth Affiliated Hospital of Soochow University, Suzhou, Jiangsu, China

**Keywords:** lipid metabolism, intracranial artery atherosclerotic stenosis, stroke, mediation, glucose

## Abstract

**Objective:**

Expanding on previous investigations, this study aims to elucidate the role of lipid metabolism disorders in the development of intracranial atherosclerotic stenosis (ICAS) and the determination of stroke risk. The primary objective is to explore the connections between lipid parameters and acute ischemic stroke (AIS), while also examining the potential mediating influence of fasting glucose levels.

**Methods:**

Retrospectively, we collected data from symptomatic ICAS patients at the First Affiliated Hospital of Soochow University, including their baseline information such as medical histories and admission blood biochemical parameters. Stenotic conditions were evaluated using magnetic resonance imaging, computed tomography angiography, or digital subtraction angiography. The associations between lipid parameters and AIS risks were investigated via multivariate logistic regression analysis.

**Results:**

A total of 1103 patients with symptomatic ICAS were recruited, among whom 441 (40.0%) suffered new ischemic events during hospitalization. After adjusting for confounding factors, the RCS curves exhibited a dose-response relationship between the atherogenic index of plasma (AIP), total cholesterol (TC), low-density lipoprotein cholesterol (LDL-C), and AIS. Further multivariate analysis revealed significant associations between these parameters and AIS. Furthermore, mediation analysis indicated that fasting blood glucose (FBG) acted as a mediator in the association between lipid parameters (AIP, TC, and TG) and AIS.

**Conclusion:**

Higher lipid parameters in ICAS patients, particularly AIP, TC, and TG, were associated with an increased AIS risk. Additionally, FBG may mediate stroke risk in ICAS patients, highlighting the need for further exploration of underlying mechanisms.

## Introduction

As a vascular disease resulting in localized lesions in the central nervous system, stroke is characterized by its high recurrence rate, significant disability incidence, and considerable economic burden (1). Acute ischemic stroke (AIS) is the most prevalent subtype of stroke, and its incidence continues to rise in China (1). Established studies have demonstrated that intracranial artery stenosis due to atherosclerosis is a notable and independent risk factor for this condition. This factor contributes to 5% to 10% of all ischemic strokes in the population and is associated with a substantial risk of stroke recurrence (2, 3). And symptomatic intracranial atherosclerotic disease is associated with a stroke recurrence risk of up to 18% within one year (3).

Lipids play crucial roles in energy metabolism, biofilm structure, and signal transduction. Disorders of lipid metabolism contribute to a range of health issues, including metabolic syndrome, obesity, and type 2 diabetes mellitus (T2DM) (4). Moreover, disturbances in lipid metabolism are central to the development of atherosclerosis (5). Irregularities in lipid metabolism not only lead to excessive lipid and lipoprotein production, which can contribute to atherogenicity, but also result in reduced levels of high-density lipoprotein cholesterol (HDL-C) and increased levels of residual and small dense low-density lipoprotein cholesterol (LDL-C) particles, among other effects (6). The relationship between lipid metabolism and glucose metabolism is complex. In addition to its impact on insulin sensitivity, hepatic glucose metabolism is involved in glycosylation reactions and is interconnected with fatty acid metabolism (7).

Previous studies have demonstrated that traditional lipid parameters such as LDL-C, HDL-C, total cholesterol (TC), and triglycerides (TG) are associated with cardiovascular disease or ischemic stroke (8–11). Specifically, LDL-cholesterol, HDL-cholesterol, and total cholesterol have been linked to intracranial carotid artery stenosis (12–14). In recent years, non-traditional lipid indicators like the atherogenic index of plasma (AIP), residual cholesterol (RC), and non-high-density lipoprotein cholesterol (Non-HDL-C) have gained attention. Several studies have shown a strong correlation with cardiovascular disease, potentially surpassing the predictive efficacy of conventional lipid markers (15–17). However, research on the relationship between these lipid parameters and intracranial artery atherosclerotic stenosis (ICAS), as well as its prognosis, remains limited. Additionally, the precise influence of glucose metabolism in this context remains unclear.

In this retrospective study, we collected demographic and clinical data from patients diagnosed with symptomatic ICAS. Our objective was to explore the relationship between lipid markers and AIS from various perspectives. Additionally, we aimed to investigate the potential mediating role of glucose metabolism, particularly fasting glucose levels, in the association between lipid metabolic parameters and AIS. This study sought to enhance our understanding of glucose-lipid metabolism and prognosis in individuals with intracranial arterial stenosis, with the ultimate goal of improving prognostic outcomes for patients affected by this condition.

## Methods

### Study population

This was a hospital-based cross-sectional study. We retrospectively analyzed data from patients with symptomatic ICAS at the Emergency Stroke Center of the First Affiliated Hospital of Soochow University (three A-level public hospital) from January 2017 to June 2022. This study involving human participants was reviewed and approved by the Institutional Review Board of the First Affiliated Hospital of Soochow University. Symptomatic ICAS was defined as AIS, transient ischemic attack (TIA), transient neurological attack (TNA; in this study, defined as any of the following transient nonfocal symptoms: disturbed consciousness, confusion, amnesia, unsteadiness, nonrotatory dizziness, positive visual phenomena, bilateral weakness or unwell feeling) (18) TIA and stroke were defined according to the tissue-based definition (AHA/ASA guidelines). Suspected patients were further confirmed by computed tomography angiography (CTA) and perfusion imaging.

The initial sample included 1612 patients. Routine blood, biochemical tests, transcranial colour Doppler sonography, cardiac ultrasound, and ECG were performed for all patients at admission. Exclusion criteria were as follows: (1) patients younger than 18 years old; (2) patients without available radiologic parameters; (3) patients who had undergone cranial surgery or intracranial endovascular treatment; (4) patients with cerebral infarction or TIA due to cardioembolic risk factors (atrial fibrillation, atrial flutter, atrial septal defect, ventricular septal defect, patent ductus arteriosus, valvular heart disease, bioprosthetic or mechanical heart valve replacement, myocardial infarction within a month, dilated myocardial infarction, sick sinus syndrome, endocarditis, etc.); (5) patients with intracranial arterial stenosis resulting from non-atherosclerotic conditions such as arterial dissection, arteritis, and Moyamoya disease, etc.; (6) patients with stroke caused by intracranial hemorrhage, subarachnoid hemorrhage, or sinus venous thrombosis.

Only 1103 patients with symptomatic ICAS were included and formed the basis of this report. In adherence with the prevailing guidelines, uniform medical guidance was provided to all patients. This included recommendations for a balanced diet, efficient blood pressure management, and appropriate medical interventions.

### Clinical information collection

We obtained data on the study population, which included demographic details, medical histories, and clinical traits. This information was sourced from electronic patient records and administrative databases. Hypertension was defined as meeting one of the following criteria: prior use of antihypertensive medication, systolic blood pressure ranging from 130 to 139 mm Hg, or diastolic blood pressure ranging from 80 to 89 mm Hg (measured on three separate occasions in a resting state). Diabetes was defined as meeting one of the following criteria: previous use of hypoglycemic drugs, fasting blood glucose ≥ 7.0 mmol/L, or postprandial blood glucose ≥ 11.1 mmol/L. Dyslipidemia was diagnosed based on criteria including LDL-C ≥ 3.4 mmol/L, TC ≥ 5.2 mmol/L, TG ≥ 1.7 mmol/L, or a history of dyslipidemia or current lipid-lowering treatments. The AIP is calculated as lg(TG/HDL−C) (19).

### Radiologic parameters

Diagnosis of intracranial stenosis was confirmed by computed tomography angiography (CTA), digital subtraction angiography (DSA), and/or high-resolution magnetic resonance imaging (HR-MRI). The diameter of stenosis (minimum lumen diameter) refers to the diameter of the residual lumen at the maximal narrowing site. Some emergency patients who were unable to undergo CTA underwent cervical and cerebrovascular ultrasonography for screening. For intracranial stenoses within the internal carotid artery (ICA), a grade of < 50% was assigned if localized flow velocity was elevated, and a grade of ≥ 50% was assigned if pre-/poststenotic flow changes were evident (18, 20). Intracranial stenoses within the ICA, middle cerebral (MCA), anterior communicating cerebral (ACA), posterior communicating cerebral (PCA), basilar (BA), and vertebral artery (VA) were classified as < 50%/≥ 50% based on established criteria as outlined in prior reports (20–22).

Patients with possible contrast allergy, asthma, renal insufficiency, hyperthyroidism, etc., who were unable to undergo CTA, underwent either HR-MRI or DSA instead after screening. When dealing with instances of multiple intracranial stenoses within a single patient, the primary stenosis was selected based on the following criteria: (1) preference was given to a symptomatic stenosis in contrast to an asymptomatic one; (2) a stenosis with a severity of ≥ 50% was prioritized over one with < 50%; (3) a singular stenosis took precedence over a tandem stenosis (18). All neuroradiological images underwent review by two independent neuroradiologists, who were unaware of the transcranial color Doppler sonography findings and clinical data.

### Evaluation of outcome

New events (AIS, TIA, and TNA) were identified through a combination of patient history, records examination, clinical evaluations, and imaging findings. The patient’s clinical symptoms were described and verified by one prehospital EMT, one emergency medicine physician, and two neurologists according to the stroke center procedure. All AIS were verified by the emergence of new neurological deficits documented in the medical records and were combined with computed tomography (CT) or MRI. The primary outcome was the occurrence of a new ischemic stroke during the hospitalization period.

### Statistical analyses

For descriptive purposes, distinctions among continuous variables were scrutinized using either the Student’s t-test or the Mann-Whitney U test, whereas discrepancies among categorical variables were evaluated using the Chi-square test. Pearson’s correlation coefficients were computed to gauge the interrelationships between the variables. Logistic regression analysis was used to identify lipid metabolism indicators associated with AIS in patients with symptomatic ICAS. In addition, the model was adjusted for gender, age, smoking history, and medical history.

Subsequently, we performed a mediating effect analysis based on the bootstrap method proposed by Preacher and Hayes through R-packet ‘mediating’ to explore whether the history of diabetes or fasting blood glucose could be modes or mechanisms by which lipid metabolism indicators influence on AIS. Statistical analysis was performed in SPSS 26.0, R programming language 4.3.0, and GraphPad Prism 9.3.0. The level of significance for these descriptive comparisons was established at 0.05 for two-sided hypothesis testing.

## Results

### Participants and their clinical characteristics

A total of 1103 patients with symptomatic ICAS were included in our study, with 441 (40.0%) of them experiencing a new ischemic stroke during the hospitalization period. The flow chart of patient enrollment is presented in **Supplementary Figure 1**.

Out of the 1,103 patients studied, 756 (68.5%) were aged 60 years or older. Additionally, 781 (70.8%) were male, with a male-to-female ratio of about 7:3. Furthermore, 194 patients (17.6%) had a history of smoking. Regarding vascular risk factors, 762 patients (69.1%) had hypertension, 320 (29.0%) had diabetes mellitus (DM), 84 (7.6%) had a history of coronary heart disease (CHD), and 84 (6.4%) had a history of hyperlipidemia. The average length of hospitalization for these patients was 9.8 days, and 1 patient (0.2%) died within two weeks. Patient baseline characteristics are shown in **Table 1**.

**Table 1:** Clinical characteristics of Symptomatic ICAS patients.

Characteristics	Total (n=1103)	With AIS (n=441)	Without AIS (n=662)	*P*
Age				<0.304
Age: 19-59, n (%)	347 (31.5%)	147 (33.3%)	200 (30.2%)	
Age: ≥ 60, n (%)	756 (68.5%)	294 (66.7%)	462 (69.8%)	
Male, n (%)	781 (70.8%)	325 (73.7%)	456 (68.9%)	0.098
Smoking, n (%)	194 (17.6%)	88 (20.0%)	106 (16.0%)	0.109
Hypertension, n (%)	762 (69.1%)	300 (68.0%)	462 (69.8%)	0.580
Diabetes, n (%)	320 (29.0%)	132 (29.9%)	188 (28.4%)	0.630
Hyperlipidemia, n (%)	71 (6.4%)	23 (5.2%)	48 (7.3%)	0.221
AF, n (%)	84 (7.6%)	16 (3.6%)	68 (10.3%)	<0.001
CHD, n (%)	84 (7.6%)	16 (3.6%)	68 (10.3%)	<0.001
FBG, median(IQR)	5.24 (4.69; 6.32)	5.26 (4.68; 6.69)	5.24 (4.69; 6.14)	0.421
TG, median(IQR)	1.28 (0.96; 1.75)	1.33 (0.99; 1.75)	1.25 (0.94; 1.76)	0.191
TC, median(IQR)	3.98 (3.25; 4.69)	4.14 (3.41; 4.85)	3.84 (3.19; 4.58)	<0.001
LDL-C, median(IQR)	2.25 (1.71; 2.91)	2.49 (1.83; 3.12)	2.10 (1.62; 2.68)	<0.001
HDL-C, median(IQR)	1.01 (0.84; 1.21)	0.94 (0.80; 1.15)	1.05 (0.88; 1.26)	<0.001
AIP, median(IQR)	0.10 (-0.06; 0.28)	0.14 (-0.02; 0.31)	0.08 (-0.09; 0.26)	<0.001

IQR, interquartile range; AIS, acute ischemic stroke; AF, atrial fibrillation; CHD, coronary heart disease; FBG, fasting blood glucose; TG, triglyceride; TC, total cholesterol; LDL-C, low-density lipoprotein cholesterol; HDL-C, high-density lipoprotein cholesterol; AIP, atherogenic index of plasma.

Of these patients, 97 (8.7%) patients presented with stenosis or occlusion of the intracranial ICA; 308 (27.9%) with MCA stenosis, 274 (24.8%) with ACA stenosis, 484 (43.9%) with PCA stenosis, and 261 (23.6%) suffered from VA or BA stenosis.

### Comparison of clinical characteristics

The participants were categorized into two groups based on whether they had AIS or not: the AIS group, which included 441 patients, and the non-AIS group, which included 662 patients. Statistical analysis indicated significant differences in CHD, atrial fibrillation (AF), TG, TC, LDL-C, HDL-C, and AIP between the two groups (*p* < 0.05, **Table 1**). However, there were no significant differences in age, gender, smoking history, hypertension, DM, fasting blood glucose (FBG), or other factors between the two groups (*p* > 0.05, **Table 1**).

**Figure 1:**
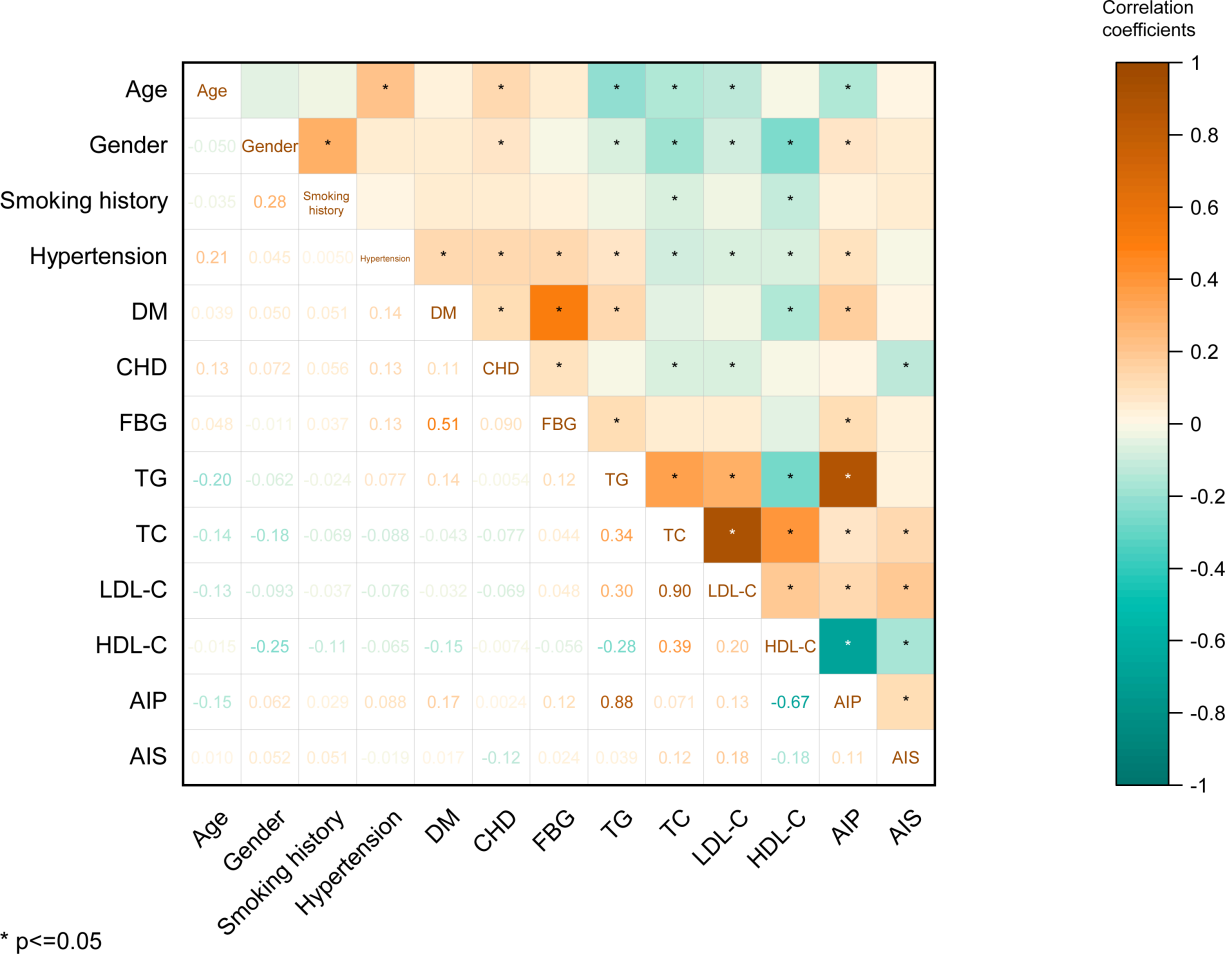
The heat map for pearson correlation coefficients between the variables.

Furthermore, we calculated the correlation coefficients between age, gender, smoking history, hypertension, DM, CHD, FBG, TG, TC, LDL-C, HDL-C, AIP, and AIS. Notably, the observed correlation coefficients revealed significant relationships: a correlation of r = 0.11 between AIP and AIS risk (*p* < 0.05), a correlation of r = 0.12 between TC and AIS (*p* < 0.05), a correlation of r = 0.12 between FBG and TG (*p* < 0.05), and a substantial correlation of r = 0.30 between LDL-C and TG (*p* < 0.05). These findings highlight that AIS risk exhibits significant yet modest associations with AIP and TC, while TG shows significant although weak correlations with FBG and LDL-C (**Figure 1**).

### Dose-response relationship of lipids parameters with AIS risk

**Figure 2:**
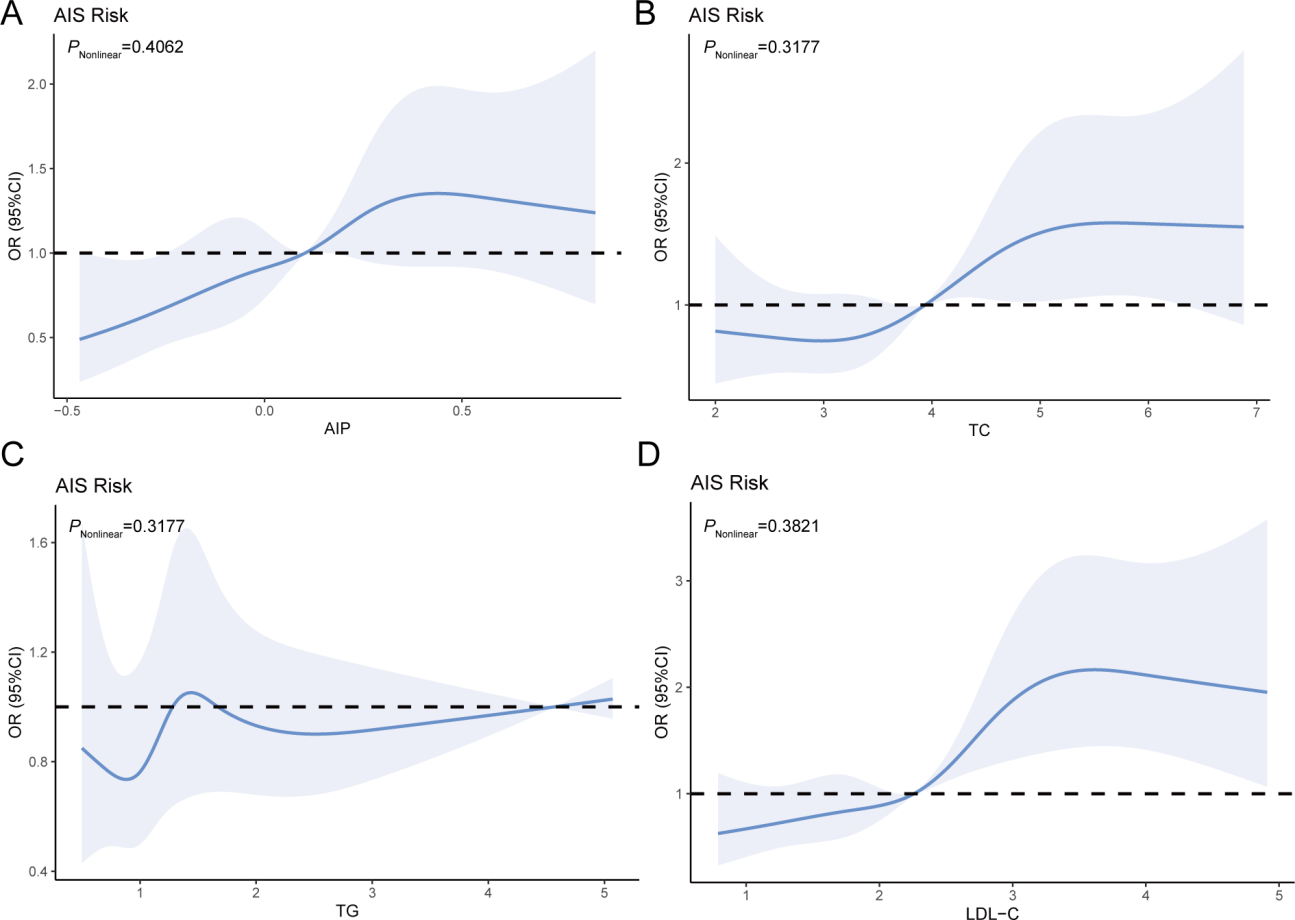
RCS analysis of the relationship between the four blood lipid parameters and AIS risk **(A–D)**. Adjusted variables: age, gender, smoking, hypertension, and CHD.

Lipid parameters were studied for their connections with AIS risk (**Figure 2**). After controlling for confounders, the RCS curves revealed a dose-response relationship with AIS. When AIP was < 0.1029, TC was < - 3.9373, or LDL-C was < - 2.2599, the plot demonstrated an increased AIS risk. However, no such associations were observed for TG (**Figure 2C**).

### Lipid parameters was associated with the occurrence of AIS

Further multivariate analysis was performed using binary logistic regression to evaluate the predictive value of lipid parameters in patients with symptomatic ICAS. For the overall sample, in univariate analyses, we found that the AIP (OR: 2.237, 95% CI: 1.424-3.512, *p* < 0.001), TC (OR: 1.248, 95% CI: 1.113-1.4, *p* < 0.001), and LDL-C (OR: 1.497, 95% CI: 1.303- 1.721, *p* < 0.001) were significantly associated with AIS status (**Table 2**). However, there were no significant associations between TG and the occurrence of AIS. These associations remained robust in multivariate analyses after adjusting for age, gender, smoking, hypertension, and CHD. We found that AIP (OR: 2.204, 95% CI: 1.387-3.502, *p* = 0.001), TC (OR: 1.269, 95% CI: 1.127-1.429, *p* < 0.001), and LDL-C (OR: 1.51, 95% CI: 1.31- 1.741, *p* < 0.001) were significantly associated with the AIS status (**Table 2**).

**Table 2:** Binary logistic regression analysis of lipid parameters for predicting the occurrence of new AIS in patients with symptomatic ICAS.

Variable	Univariate OR	*P*	Multivariate OR	*P*
AIP	2.237 (1.424, 3.512)	< 0.001	2.204 (1.387, 3.502)	0.001
TC	1.248 (1.113, 1.4)	< 0.001	1.269 (1.127, 1.429)	< 0.001
TG	1.068 (0.959, 1.189)	0.233	1.066 (0.956, 1.19)	0.249
LDL-C	1.497 (1.303, 1.721)	< 0.001	1.51 (1.31, 1.741)	< 0.001

These factors were adjusted in the multivariate regression analysis: age, gender, smoking, hypertension,and CHD.

CHD, coronary heart disease; TG, triglyceride; TC, total cholesterol; LDL-C, low-density lipoprotein cholesterol; AIP, atherogenic index of plasma.

### Mediating roles of FBG

**Figure 3:**
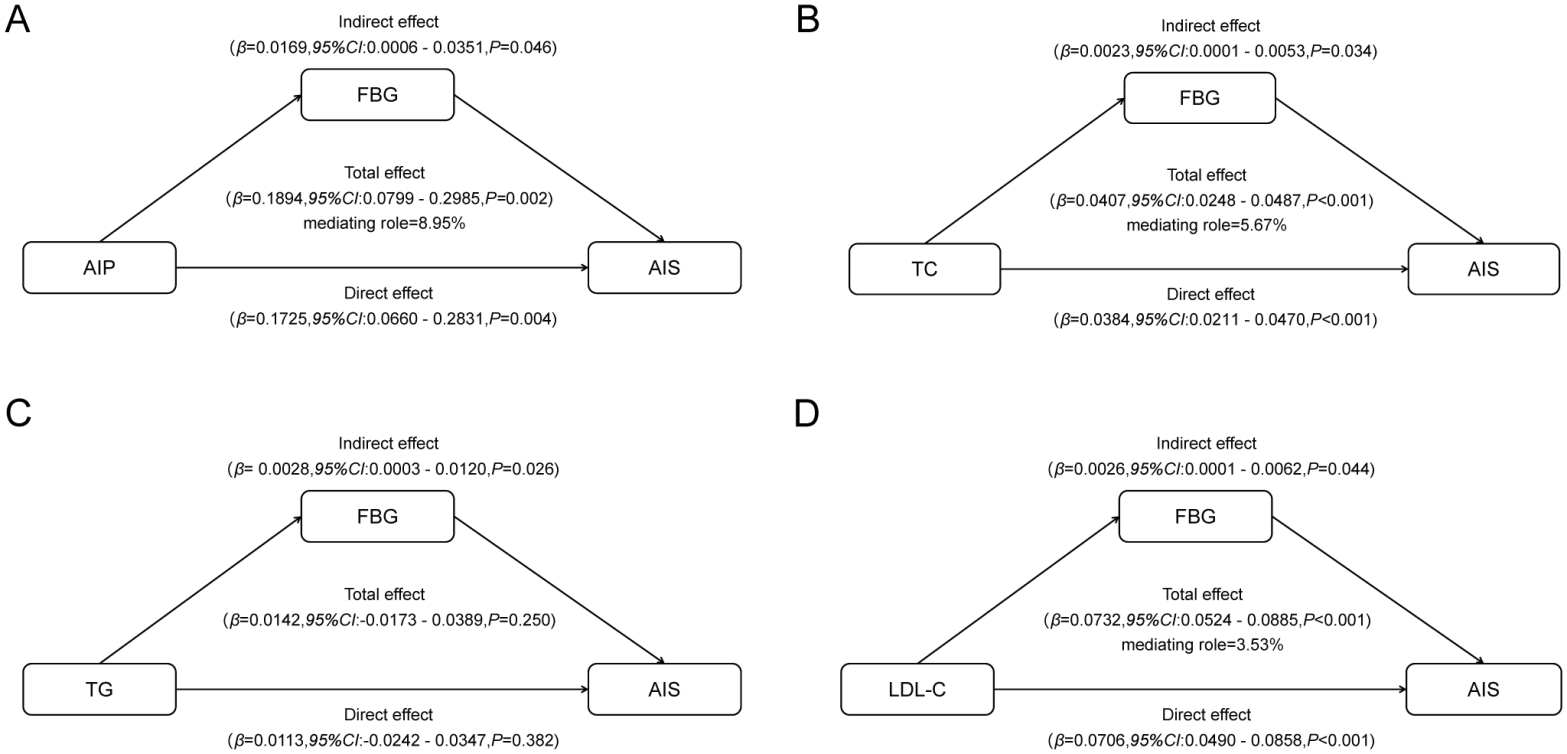
Mediated associations between four blood lipid parameters and AIS risk by fasting blood glucose: **(A)** mediated associations between AIP and AIS risk by fasting blood glucose; **(B)** mediated associations between TC and AIS risk by fasting blood glucose; **(C)** mediated associations between TG and AIS risk by fasting blood glucose; **(D)** mediated associations between LDL-c and AIS risk by fasting blood glucose.

A mediation analysis was performed to explore whether FBG mediates the relationship between lipids parameters and AIS in patients with symptomatic ICAS (**Figure 3**).

As predicted, FBG was found to be a significant mediator. The indirect effect of FBG on the association between AIP and AIS implied a 0.0169-point increase in the likelihood of AIS (95% CI, 0.0006 - 0.0351), accounting for approximately 8.95% of the association between AIP and AIS. The persistent significance of FBG as a mediating factor extends to traditional lipid parameters as well. The indirect effect of FBG on the association between TC and AIS implied a 0.0023-point increase in the likelihood of AIS (95% CI, 0.0001 - 0.0053), accounting for approximately 5.67% of the association between TC and AIS. Similarly, the indirect effect of FBG on the association between LDL-C and AIS implied a 0.0026-point increase in the likelihood of AIS (95% CI, 0.0001 - 0.0062), accounting for approximately 3.53% of the association between LDL-C and AIS. However, we did not observe a significant mediating effect of FBG on the association between TG and AIS risk.

## Discussion

In this study, we conducted a comprehensive analysis to investigate the interplay among lipid parameters, FBG, and AIS in patients diagnosed with symptomatic ICAS. The principal findings of this study can be summarized as follows: (1) AIP, TC, and LDL-C exhibited positive associations with AIS in symptomatic ICAS patients; (2) FBG played a mediating role in the occurrence of AIS, suggesting a potential link between AIS and glucose metabolism.

Given the substantial prevalence of ICAS in Asian populations and its potentially severe consequences, there is an urgent need to investigate its pathogenesis, as well as the factors and mechanisms that influence it (23, 24). Abnormal brain perfusion is associated with changes in the expression of several genes, including ADM, BDNF, CDKN1A, CREB, GADD45G, IL6, nNOS, and TM4SF1 (25). Most of these genes are linked to inflammation, oxidative stress, and apoptotic processes, and some have been identified as potential blood markers of cerebral ischemia (25–27). The significant involvement of lipid and glucose metabolism in these processes suggests the potential to influence the impact of transcriptome expression profiles on characterizing molecular processes activated in the induction of neuroprotective mechanisms. However, relevant data in this regard are still limited (28, 29).

Lipids play a pivotal role in both the progression and the localization of the disease and exhibit a substantial association with its prognosis (30, 31). In our study, a larger proportion of patients exhibited PCA stenosis, possibly due to the higher likelihood of severe stenosis and associated symptoms in patients with PCA stenosis (32). The progression from intimal thickening and atherosclerotic plaque formation to ICAS and, ultimately, AIS involves several stages, with lipid metabolism and inflammation playing key roles (31, 33). Previous studies have linked hyperlipidemia and high LDL-C levels to an increased risk of ICAS (34). Lowering LDL-C levels has been a primary focus of therapy to prevent ischemic stroke (34). Research suggests that high-dose statin therapy effectively stabilizes symptomatic intracranial atherosclerotic plaques, as shown by HR-MRI, and leads to a significant reduction in LDL levels (35). Additionally, intensive lipid-lowering therapy with statins has shown promise in reducing the extent of stenosis in asymptomatic ICAS patients (36). While evidence suggests that LDL-C may play a role in ICAS pathogenesis and progression, there is some debate. Mendelian analysis indicates that genetic variants related to LDL-C have a relatively modest impact on AIS (37). The Chinese Intracranial Atherosclerosis (CICAS) study have not consistently supported a significant correlation (38). However, another Chinese large retrospective cohort study demonstrated a concentration-dependent relationship between LDL-C levels and the asymptomatic ICAS prevalence (39). This variability may be attributed to natural fluctuations in LDL-C levels. Our study’s data analysis supports a strong association between LDL-C levels during acute vascular events and AIS development, consistent with existing literature.

Compared to other non-traditional lipid parameters, AIP stands out as an important and significant independent risk factor for both intracranial and extracranial atherosclerotic stenosis (16). It reflects the particle size cutoff between two phenotypes of LDL-C particles, A and B, indirectly indicating levels of small dense LDL cholesterol (sdLDL-C) (19). In comparison to LDL-C levels, sdLDL-C levels are poised to serve as a superior indicator of atherosclerotic cardiovascular disease (40). Due to its low receptor affinity and small particle size, sdLDL-C is more prone to contributing to atherosclerosis formation (40, 41). While sdLDL-C assays have limited clinical utility, AIP offers a simplified means of assessing sdLDL-C levels (40, 42). Our results demonstrated that AIP independently associated with the risk of AIS in ICAS patients and exhibited a dose-response relationship.

Based on previous population studies and our data analysis, no statistically significant correlation has been observed between TG and the development of ICAS or AIS (43). However, TG, as an essential biomarker of lipid metabolism, is closely associated with the pathogenesis of abnormal glucose metabolism, including insulin resistance (44). In muscle and liver tissues, intracellular fat accumulation, particularly glycerol diacyl, impairs the insulin signaling pathway, leading to elevated blood glucose levels and the development of T2DM (45). Lipid metabolism and glucose metabolism exhibit complex interrelationships. The intima-media thickness of the common carotid artery in patients with TG carotid atherosclerosis is strongly correlated with a higher triglyceride-glucose (TyG) index, serving as a simple surrogate for insulin resistance, and is independently associated with stroke (46, 47). On the other hand, blood glucose is intricately linked to the pathogenesis of ICAS and AIS, as well as prognosis. Recent studies have identified a significant association between blood glucose fluctuations and severe internal carotid artery stenosis in patients with T2DM (48). Additionally, stress-induced hyperglycemia heightens the risk of stroke recurrence in patients with ICAS (49). Our analysis demonstrated that FBG, rather than a history of DM, mediated the positive association between lipid metabolism indicators (AIP, TC, and LDL-C) and the risk of AIS in patients with ICAS. This underscores the significance of rigorous fasting glucose control in patients with ICAS. Dysfunctions in glucose and lipid metabolism likely play a central role in the broader pathogenesis of atherosclerosis, although further investigation is needed to elucidate the precise mechanisms.

The interpretation of our data should be tempered by consideration of the study’s limitations. The recruitment of participants from a single clinical unit and the exclusion of asymptomatic multiple stenoses in this study may impact the generalizability of the findings to a broader population. The patients underwent examinations according to the stroke center’s protocols, and no tests for glycosylated hemoglobin or additional blood markers for assessing cerebral ischemia were conducted (although some patients received further blood tests after being admitted to the hospital). Another limitation of this study is the absence of further stratification among patients based on different stenosis sites and degrees. Given that the study cohort consisted of symptomatic ICAS patients, many of whom presented with multiple stenoses, additional analysis was foregone as further utilization of diverse imaging methods (DSA, CTA, and MRA) for plaque characterization might introduce unwarranted heterogeneity. Furthermore, as a cross-sectional observational study, we did not explore the underlying mechanisms associated with FBG throughout the entire pathogenesis process. Therefore, specific clinical implications require further investigation.

## Conclusion

In summary, our data reveal significant positive associations between the AIP, TC, and LDL-C with the occurrence of AIS in patients presenting with symptomatic ICAS. Notably, our findings underscore the mediating role of FBG in AIS development. These insights contribute to a more profound understanding of the intricate interactions between lipid metabolism and glucose homeostasis, warranting further investigation into their clinical implications and underlying mechanisms.

## Data Availability

The original contributions presented in the study are included in the article/**Supplementary Material**. Further inquiries can be directed to the corresponding author/s.
