# Supplementary figures and images for: Lipid on stroke in intracranial artery atherosclerotic stenosis: a mediation role of glucose
Source: Front Endocrinol (Lausanne). 2024 Aug 20;15:1322114. doi: 10.3389/fendo.2024.1322114 (PMC11368875; doi:10.3389/fendo.2024.1322114)

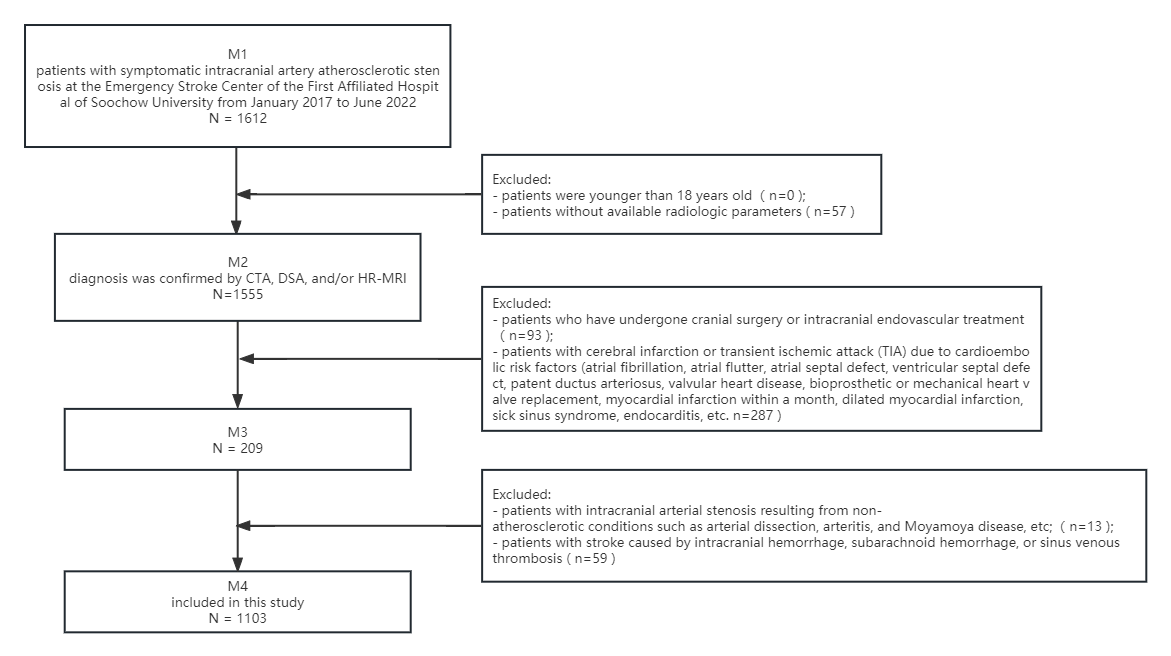

Supplement: Supplementary file 1 [file Image1.png]
